# Treatment of Hydrocele

**Published:** 1848

**Authors:** L. Dunlap


### ARTICLE VI.

*Treatment of Hydrocele*—a paper read before the Indianapolis Medical Society. By L. Dunlap, M.D.

Mr. President:—I propose, this evening, to make a few remarks upon the radical cure of hydrocele of the tunica vaginalis. It is not my purpose to advance any new theory or doctrine, but to improve upon the many modes of operating and dressing, that have been adopted by eminent Surgeons, for ages past, and to point out some peculiarities, which I have found advantagious and successful.

I will, in the first place, call to mind, two of the most prominent modes of treatment and my objections to them. We are well aware that every variety of treatment has occasionally failed, in the most experienced hands; but it is not surprising, when we take into consideration, the infinite variety of conditions, which the structures of the human body may assume.

The tunica vaginalis, being a process of the peritoneum which invests the testicle, in a natural state secretes a fluid, which lubricates the surface of the organ; but in a morbid condition of the vessels, either the secretory or absorbent, (the latter case being exceedingly rare,) an accumulation of fluid, is the consequence. Dropsical swellings are generally the result of an increased secretion from the arteries; and in some instances, the services of a physician are only necessary; but in a great majority of cases, we are compelled to resort to other means, and call in the aid of a surgeon. I will here observe, that what I am about to say, is in relation to the treatment of adults. For the hydrocele of children, I have, in every instance, been pleased to see recover by the use of a little alterative medicine, and by time.

We are told, that merely drawing off the water from the tumor, will not produce a cure, for it will readily accumulate again. This is true, as a general rule; although, in some instances, a cure has been effected in this way; but it is of rare occurrence.

It seems to be the general impression, that it is necessary, in effecting a radical cure, that the obliteration of the cavity be effected. This, I believe to be the fact, in most cases; yet there may be a condition that will produce the desired effect, without resorting to this ultimatum.

A distinguished surgeon has said that inflammation induces a suppression of the action of the secretory vessels, which pour out the fluid, even when the degree of inflammation is insufficient to produce adhesions and consequent obliteration of the cavity.

It is easy to discover that the real legitimate mode or indication of treatment is to place the diseased vessels in their healthy condition; but so intricate is our organization, its peculiar surfaces, and ten thousand impressions from the nervous system, that it is an impossibility to indicate the constitutional treatment, or the real amount of inflammation necessary to produce the desired results.

The operations which have been resorted to are by seton, caustic, injection, and incision. I shall pass by the two first, as they are nearly obsolete, and dwell only on the two last, injection and incision. The first is said to excite inflammation, and the latter to fill the cavity with granulations.

We will examine the operation by injection, which is of so dubious a character as always to entertain doubts of success. I will give the words of a celebrated surgeon in France: "The spirit of wine was used, which produced a cure, but the inflamation was so violent that he afterwards tried a milder injection, which consisted of wine." Lambert published at Marseilles, early in the seventeenth century, advising injections of corrosive sublimate in lime water, and he has related cases of success. Mr. Sharpe also made trial of spirits of wine, which cured the hydrocele, but not without causing dangerous symptoms, and subsequent abscesses in the scrotum. Zinc and iodine have been used with about the same results.

Now, from the admission of these celebrated surgeons, the operation by injection is very uncertain, for at times the injection may be too strong, so as to endanger the life and happiness of the patient; and at others too weak, so that it will be necessary to repeat the operation, which is a tedious delay, to say the least of it. From the peculiarities in constitution, it is impossible to precisely fit the injection to suit the case; and no human foresight can form a scale for them.

The other mode is by incision. Sir Astley Cooper describes the operation thus:

"This is done by beginning an incision at the upper part of the swelling, and extending it two thirds downwards; for if it be made to the lower part of the tunica vaginalis, it leaves the testis too much exposed; and produces excessive inflammation in it. The water being evacuated, and the state of the testis learned, as well as if there be any disease connected with it (as cysts on the testis), a little common flour is sprinkled in, and thus the surface is forced to granulate; and any return of the disease is sure to be prevented. Very seldom is such an operation required, and it ought not to be resorted to but in cases of great doubt with respect to the disease, as it is one of great severity. After this operation, a poultice only should be applied; and the cure is effected by suppuration and granultation."

Sir Astley Cooper is very high authority indeed, yet we think that no human is infallible; and, as inexperienced as we are, we claim views of our own on all subjects that may enlist our attention. We will describe the mode of operating and dressing, which we think is entitled to some consideration.

The first thing to be considered is, to put your patient's system in a healthy condition with Abernethian care; when this is accomplished, you may proceed to the operation, which is as follows: Commence the incision at the superior part of the tumor, making it through the integuments to the most dependent part—then the inferior portion of the tumor is punctured with a thumb lancet; and a director immediately introduced to guide a bistoury, which is carried up to the superior part of the tumor. The body of the testicle is now examined, and if cysts are found on its surface, clip them with the scissors, and the operation is completed. All that remains is the dressing.

We take a narrow piece of white lute string ribbon, and introduce it on the point of a probe, extending it under the tunica vaginalis, superiorly as far as the probe will extend, then crowding a portion of the tent on each side of the testis, and cover the part with lint—apply a compass and the suspensory bandage, and send the patient to bed. Let the tent remain till it produces considerable pain and complaint from the patient, which will be in from six to eighteen hours—then take hold of the end of the tent, which is left without the wound, and draw it out, which is done without the least difficulty, and continue the lint and the bandage until the patient is well.

Now the advantages in this mode of operating you will readily perceive. The incision is free and will not allow any fluid, be it blood or serum, to accumulate at the bottom of the scrotum, which might, in such an event, be very troublesome. The cysts, if there should be any, can be seen and clipped with the scissors, which destroys them effectually; and the tent of lute string ribbon, has an advantage over all tents that I have ever used; as you can send it to any place on the end of your probe, with unerring certainty, and withdraw with the utmost facility. This species of tent, is a decided improvement, and we would recommend the faculty to test its value by use. Another advantage is, we can regulate the quantum of inflammation necessary, by the irritation it produces, and withdraw it at any moment; but if we use flour, and do not carry the incision to the most dependent part, we will have much difficulty in getting rid of it when we wish.

The danger of inflammation that Sir Astley Cooper is so fearful of, in consequence of a free incision, need not be apprehended in this climate, and particularly so, if due care is taken to have the system well prepared for the operation. Inflammation, no doubt, might be dreaded in England; but I consider that it is much modified in its violence, in all countries abounding in malaria.

Should it so happen that you have too great an amount of inflammation, you can subdue it by the timely use of emetics, by the administration of one or two per day, as the urgency of the case may seem to demand.

